# How multimorbidity and socio-economic factors affect Long Covid: Evidence from European Countries: Piotr Wilk

**DOI:** 10.1093/eurpub/ckac129.137

**Published:** 2022-10-25

**Authors:** P Wilk, M Ruiz-Castell, V Moran, M Noel Pi Alperin, T Bohn, G Fagherazzi, M Suhrcke

**Affiliations:** 1 Department of Epidemiology and Biostatistics, Western University, London Ontario, Canada; 2 Institute of Social and Preventive Medicine, University of Bern, Bern, Switzerland; 3 Department of Precision Health, Luxembourg Institute of Health, Luxembourg, Luxembourg; 4 Living Conditions, Luxembourg Institute of Socio-Economic Research, Luxembourg, Luxembourg

## Abstract

**Introduction:**

An increasing number of individuals continue reporting symptoms following the acute stage of Covid-19 infection. Few studies have investigated the factors related to Long Covid. Our aim was to assess how multimorbidity, socio-economic factors (immigration, education, employment, and income), and country of residence affect the presence and number of persistent symptoms attributable to Covid-19 illness in Europe.

**Methods:**

We used data from the SHARE Corona surveys collected in 2020 and 2021. The sample included 4,004 respondents aged 50 years and older who were affected by the Corona virus. The outcome was the number of persistent symptoms attributable to Covid-19 illness, including: fatigue; cough, congestion, shortness of breath; loss of taste or smell; headache; body aches, joint pain; chest or abdominal pain; diarrhoea, nausea; and confusion. We conducted a multilevel analysis for a hurdle model with negative binomial distribution.

**Results:**

Overall, 73% of respondents were estimated to have at least one persistent symptom associated with Covid-19 illness and, on average, they had 2.73 symptoms. However, there were some statistically significant across country differences in the presence and number of symptoms. Respondents who were employed were more likely to report at least one symptom (OR = 1.40) and those with higher levels of education were less likely to report any symptoms (OR = 0.67). Respondents with multimorbidity had an increased risk of experiencing an additional symptom (RR = 1.12) while respondents who were employed had a decreased risk of experiencing an additional symptom (RR = 0.85).

**Discussion and conclusions:**

Presence and number of persistent symptoms associated with Covid-19 illness was highly prevalent and varied significantly across European countries. Evidence from the present work underscores the need to target high-risk groups and those with multimorbidity to reduce long-term health consequences of Covid-19.

